# Comparative cadaveric study of needle and conventional arthroscopy techniques of the feline stifle joint

**DOI:** 10.1177/1098612X251410842

**Published:** 2025-12-15

**Authors:** Chiara Anna Koecher-Vodnarek, Julia Helm, Eva Schnabl-Feichter

**Affiliations:** Small Animal Surgery, Department for Companion Animals and Horses, University of Veterinary Medicine Vienna, Austria

**Keywords:** Stifle joint, needle arthroscopy, conventional arthroscopy, cadaveric study, articular cartilage injury

## Abstract

**Objectives:**

Arthroscopy is a well-established diagnostic and therapeutic method for canine stifle pathology; however, its use in cats remains under-reported. This study aimed to evaluate and compare the efficacy and safety of conventional arthroscopy (CA) and needle arthroscopy (NA) in feline stifles.

**Methods:**

Paired stifles from 20 feline cadavers without clinical or radiographic stifle pathology were randomly assigned to two equal groups: CA (1.9 mm, 30°) and NA (1.9 mm, 0°). Arthroscopy was performed by a board-certified surgeon using a three-portal method and predefined evaluation sequence, ending with medial meniscus assessment. In 10 randomly selected stifles from each group, an extra-articular distractor was applied before medial meniscus evaluation. Procedure duration, visualisation quality, surgical difficulty and cartilage lesions were recorded. Periarticular and iatrogenic articular cartilage injuries (IACIs) were assessed via dissection and India ink staining.

**Results:**

NA yielded a lower mean surgical difficulty score, shorter mean arthroscopy duration and higher rate of complete medial meniscus visualisation than CA. No significant differences were found in absolute IACI (CA with distraction [CA-D]: 4.4 ± 2.8 mm^2^; CA without distraction [CA-nD]: 5.4 ± 2.4 mm^2^; NA with distraction [NA-D]: 3.9 ± 2.0 mm^2^; NA without distraction [NA-nD] 3.6 ± 2.4 mm^2^) or in percentage surface area affected (CA-D: 1.0 ± 0.6%; CA-nD: 1.3 ± 0.6%; NA-D 0.9 ± 0.4%; NA-nD 0.9 ± 0.6%).

**Conclusions and relevance:**

Stifle arthroscopy was feasible using both conventional and needle arthroscopes in feline cadavers without stifle pathology and may be considered a minimally invasive tool for diagnosing feline stifle disease. NA in smaller patients may offer advantages over CA in terms of feasibility and procedure duration. Although not statistically significant, NA tended to result in fewer IACIs. IACIs per joint were comparable to values reported in dogs.

## Introduction

Canine stifle disease has been well studied, while feline stifle conditions remain under-researched.^1–8^ Medial meniscus injuries, observed in 47–67% of feline stifles with cranial cruciate ligament (CCL) deficiency,^2,7,8^ resemble those seen in dogs,^9^ highlighting the need for joint exploration. Arthroscopy has become a well-established tool for canine stifle disorders,^10^ with joint distraction enhancing procedural success.^11–14^ In contrast, reports of feline stifle arthroscopy are limited,^4,15–19^ with applications confined to osteochondritis dissecans,^15^ patellar disorders^16^ and suspected meniscal injuries.^4^ Because of the smaller joint size, conventional arthroscopy (CA) in cats can be challenging and may increase the risk of iatrogenic articular cartilage injury (IACI), whereas needle arthroscopy (NA) may improve procedural feasibility.^19^ In canine stifles, NA allows rapid, low-morbidity assessment with high sensitivity for detecting meniscal tears.^20^ Only one case report has described NA use in a cat: for treating shoulder dysplasia.^21^ The current literature lacks both a detailed account of feline stifle arthroscopy and comparative studies of available techniques. This study aimed to evaluate the feasibility, efficacy and safety of CA and NA, with and without distraction, in feline stifles, and to identify procedural aspects relevant to clinical application. We hypothesised that both techniques would be feasible, with NA offering improved usability and reduced IACI compared with CA.

## Materials and methods

### Specimens

A total of 40 stifles from 20 feline cadavers euthanased for unrelated reasons were thawed for 48 h before use. Specimens were included based on skeletal maturity and the absence of considerable clinical or radiographic stifle pathology. Cadavers with degenerative joint disease scores (DJDS)^22^ of 1.5 and above were excluded. Body weight, breed and sex were recorded. Using an online randomisation tool (PiliApp Software), the stifles were assigned to equal groups of NA (n = 20) and CA (n = 20), with half of the stifles in each group (n = 10 per group) randomised to distraction. A pilot cadaver was dissected to assess anatomy and positional relationships, and the long digital extensor tendon and cruciate ligaments were noted as structures at potential risk for injury during arthroscopy.

### Procedure

Cadavers were positioned in dorsal recumbency with pelvic limbs extended over the table. Arthroscopies were performed by the same boarded surgeon and assistant using a three-portal technique. Joints were distended with irrigation fluid via a 20 G needle inserted dorsomedial to the patella, which subsequently served as an egress port.

**Figure 1:**
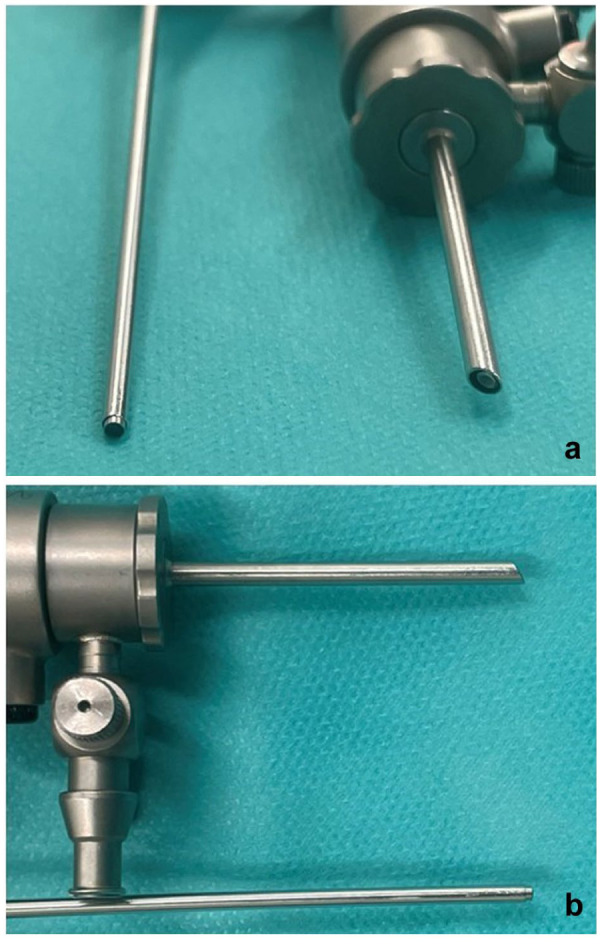
Comparison of the 1.9 mm 30° conventional arthroscope tip with its 3.0 mm cannula and the 1.9 mm 0° needle arthroscope tip with its 2.4 mm cannula (all instruments: Arthrex) used for stifle arthroscopy in feline cadavers. (a) A frontal view with the 30° arthroscope on the right. (b) A sagittal view with the 30° arthroscope positioned in the upper portion of the frame

The craniolateral portal was created with a stab incision (#11 blade) lateral to the patellar tendon at the junction of the proximal and middle thirds of the distance between the apex patellae and the proximal tibial tuberosity, and widened using a mosquito clamp. Cannulas (3.0 mm for CA [AR-3372-1902], 2.4 mm for NA [AR-3210-0050]; Arthrex) with blunt obturators (2.5 mm for CA [AR-3375-1902], 1.9 mm for NA [AR-3210-0050]; Arthrex) were inserted with the joints flexed to 90° and advanced into the lateral pouch upon extension before being replaced with the arthroscopes (1.9 mm 30° with 80° field of view for CA [AR-3210-0043], 1.9 mm 0° with 120° field of view for NA [AR-3350-193]; Arthrex) (Figure 1). Fluid ingress was managed using an arthroscopy pump (DualWave Arthroscopy Fluid Management System) set at 30 mmHg and gradually adjusted. A craniomedial instrument portal was established similarly, medial to the patellar ligament.

**Figure 2:**
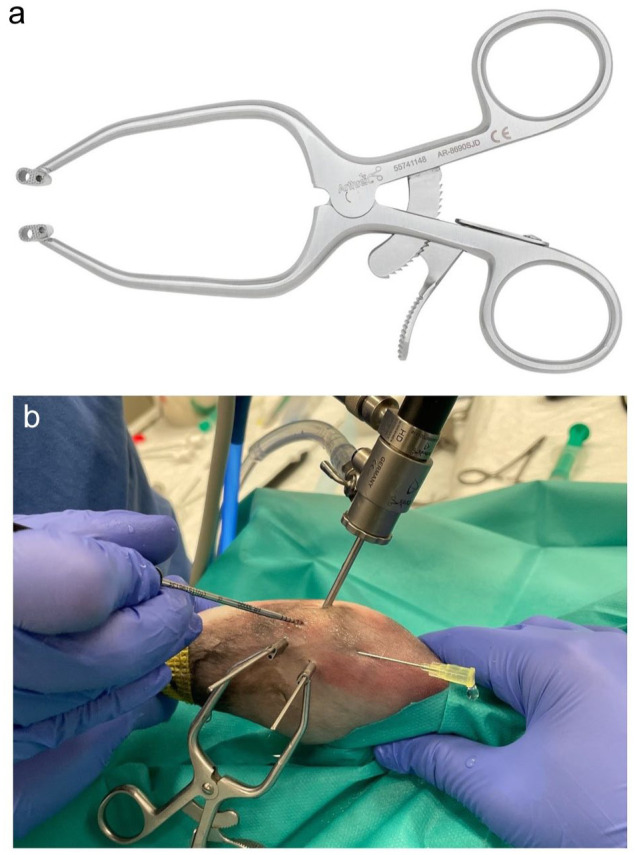
(a) Image of the small extra-articular joint distractor (Arthrex) employed in stifle arthroscopy procedures on feline cadavers. (b) Photograph of an arthroscopic procedure performed on a right feline cadaveric stifle using a 1.9 mm 30° arthroscope and a 3.0 mm cannula inserted via the craniolateral portal. A 20 G needle served as the proximomedial egress port, and a joint hook probe was inserted via the craniomedial portal. For joint distraction, 1.6 mm Kirschner wires were placed medially: one in the central femoral condyle at the level of the fabella and another in the caudoproximal tibia at the level of the proximal tibial tuberosity. The extra-articular stifle distractor was applied before medial meniscus assessment

In distracted cases, 1.6 mm Kirschner wires were placed medially in the central femoral condyle at the fabellar level, and in the caudoproximal tibia at the proximal tibial tuberosity level, as previously described,^12^ before applying the small extra-articular distractor medially (AR-8690SJD) (Figure 2).

Intra-articular structures were examined in the following order: trochlear groove and ridges, patella, cruciate ligaments, long digital extensor tendon, lateral femorotibial joint and meniscus (with probing), medial femorotibial joint and meniscus (with probing). The proximal compartment was assessed with the stifle extended and the egress port closed to maintain maximal distension. Meniscal evaluation included joint flexion, internal rotation and varus stress for the lateral meniscus, and external rotation with valgus stress for the medial meniscus. A small joint hook probe (3.2 mm [AR-30000]) was used for palpation. Fat pad obstruction was managed by distal retraction or portal repositioning.

**Figure 3:**
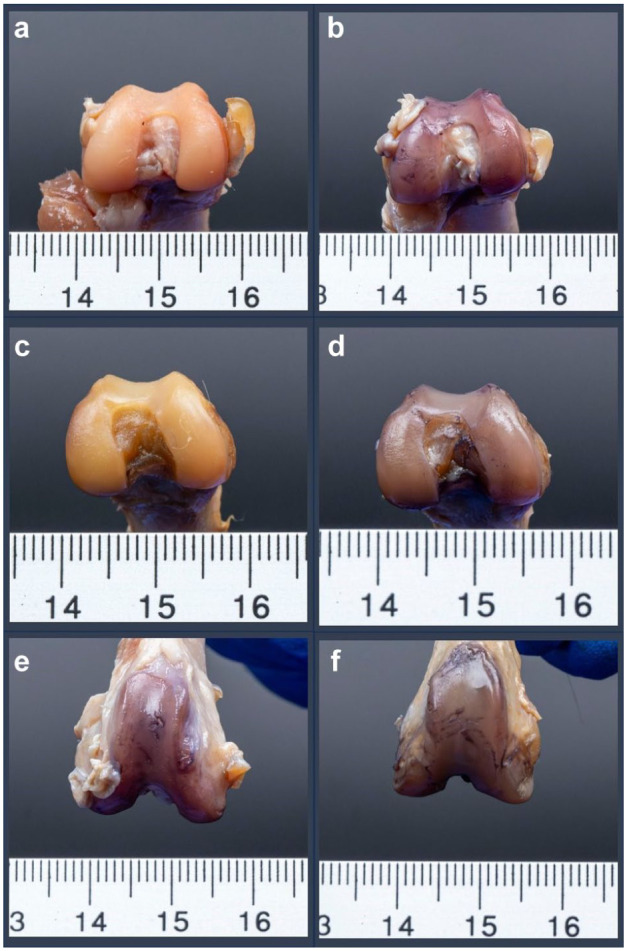
Representative scale-marked photographs of dissected feline femoral articular surfaces obtained from cadaveric stifle joints after (a,b,e) right-sided 1.9 mm 30° conventional arthroscopy and (c,d,f) left-sided 1.9 mm 0° needle arthroscopy. Articular surfaces are displayed before staining (a,c) and after cartilage staining with an India ink assay (b,d–f)

After arthroscopy, the stifles were dissected and IACI was assessed using an India ink assay.^23,24^ Scale-marked photographs of all articular surfaces were taken before and after staining (Figure 3).

### Arthroscopic outcome measures

Feasibility, structural visualisation, meniscal palpation and perioperative difficulties were recorded. Meniscal visualisation was scored on a 3-point scale (1 = 100%, 0.5 = 50–99%, 0 = <50% visualised), and articular cartilage was graded according to the Modified Outerbridge (MOS) classification.^25^ Procedures were timed, and surgical difficulty was rated using a previously published 3-point scale (Table 1).^10^

**Table 1:** Grading criteria for surgical difficulty during stifle arthroscopy, based on a 3-point scale (1 = low, 3 = high) as described by Cortés et al^10^

Surgical difficulty score	Definition
1	Single joint insufflation Single attempt for portal placement No cannula obstructing visualisation No palpable or visible IACI
2	One or more cannula(s) replaced one to three times Multiple insufflation attempts Loss of fluid ingress Cannula partly obstructing the view
3	One or more cannula(s) replaced more than three times Palpable or visible IACI Cannula completely obstructing the view

IACI = iatrogenic articular cartilage injury

### Dissection outcome measures

Stifles were examined for peri- and intra-articular injuries. IACI location and area were identified using ImageJ software (US National Institutes of Health, Bethesda, MD). The percentage of cartilage retaining ink was calculated relative to the total cartilage area. Outcome analysis was performed blinded to specimen identity.

### Statistical analysis

The minimal sample size per group was 10 (alpha = 0.05; 1-beta = 0.8). Continuous data are presented as mean ± SD (range), and ordinal data as median and range. Normality was assessed using the Shapiro–Wilk test. Mann–Whitney U-tests were applied for comparisons between two groups, and Spearman’s rank correlation for associations between variables. For comparisons among the four subgroups, Kruskal–Wallis tests were applied, with Holm adjustment for post hoc pairwise analysis. Statistical significance was set at *P* <0.05.

## Results

### Specimens

The study included nine female and 11 male domestic shorthair cats with a mean weight of 3.4 ± 1.0 kg (range 2.3–6.3), with no significant clinical or radiographic stifle abnormalities and a mean DJDS of 0.7 ± 0.7 (range 0.0–1.4).

### Arthroscopic outcome measures

**Figure 4:**
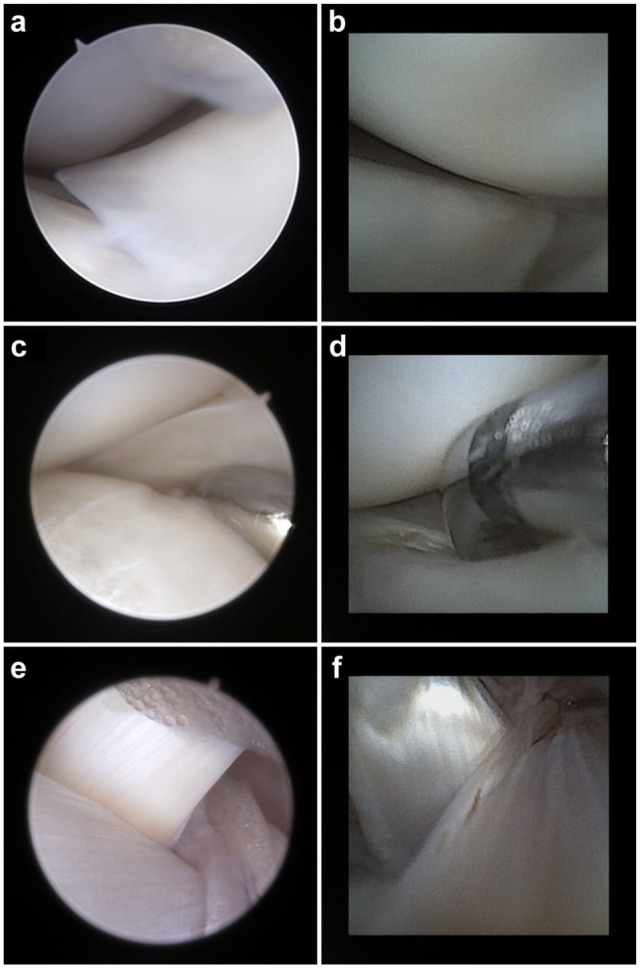
Representative intra-articular views of the (a) right and (b) left medial meniscus, (c,d) meniscal palpation and (e,f) cruciate ligaments obtained from feline cadaveric stifle joints using (a,c,e) 1.9 mm 30° conventional arthroscopy and (b,d,f) 1.9 mm 0° needle arthroscopy

Complete or partial evaluation of intra-articular structures was feasible in all specimens. Representative intra-articular images obtained with NA and CA are provided in Figure 4.

Full visualisation of all predefined structures and palpation of both menisci was achieved in six (30%) stifles in the CA group and eight (40%) in the NA group.

Optimal arthroscopic visualisation was obtained at irrigation pressures in the range of 35–40 mmHg during NA and 30–35 mmHg during CA. The overall median MOS was 2 (range 0–3), with no difference between groups (*P* = 0.7).

Complete visualisation of the medial meniscus was achieved in nine (90%) and eight (80%) stifles in the NA-D and NA-nD groups, while the lateral meniscus was completely visualised in six (60%) and five (50%) stifles, respectively. In both CA groups, complete medial and lateral meniscus visualisation was obtained in six (60%) stifles each. In the remaining procedures, meniscal visualisation was classified as 50–99% visualised. Medial meniscus visualisation was significantly better in the NA group compared with the CA group (*P* = 0.03), while visualisation of other structures and overall visualisation did not differ between groups.

Surgical difficulty scores were lower in the NA group (*P* = 0.02), with a median score of 1 (range 1–2), compared with 2 (range 1–3) in the CA group. The most common difficulty was fat pad obstruction, observed in 90%, 80%, 70% and 50% of cases in the CA with distraction (CA-D), CA without distraction (CA-nD), NA with distraction (NA-D) and NA without distraction (NA-nD) groups, respectively. Distal retraction of the fat pad was sufficient in all but four stifles, which required more proximal portal replacement. There was a significant association between surgical difficulty and overall visualisation, medial and lateral meniscus visualisation and procedure duration across all subgroups (*P* <0.01–0.04).

**Figure 5:**
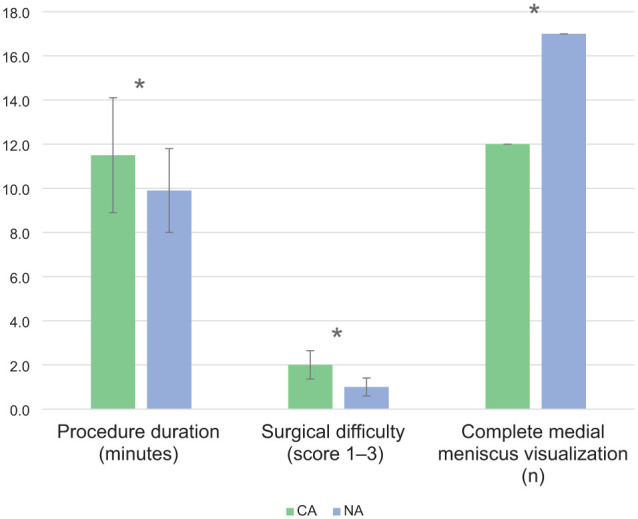
Comparison of conventional arthroscopy (CA) and needle arthroscopy (NA) in cadaveric feline stifle joints. Procedure times were significantly shorter in the NA group (mean 9.9 ± 1.9 mins, range 6.5–13.7) compared with the CA group (mean 11.5 ± 2.6 mins, range 6.3–15.3) (*P* = 0.03). Surgical difficulty, scored on a 3-point scale, was lower in the NA group (median 1, range 1–2) than in the CA group (median 2, range 1–3) (*P* = 0.02). Medial meniscus visualisation was superior in the NA group, with complete visualisation achieved in 17/20 (85%) stifles, compared with 12/20 (60%) in the CA group (*P* = 0.03). The asterisk (*) indicates statistically significant differences between groups (*P* <0.05)

Procedure times were shorter with NA (mean 9.9 ± 1.9 mins, range 6.5–13.7) compared with CA (mean 11.5 ± 2.6 mins, range 6.3–15.3) (*P* = 0.03) (Figure 5). Pin placement and application of the extra-articular stifle distractor took a mean of 35 ± 8.5 s (range 24–57), not significantly contributing to procedure duration.

Visualisation outcomes, scoring distributions and procedure times are presented in Table 2 and Figure 5.

**Table 2:** Arthroscopic findings in 40 feline cadaveric stifles assigned to needle arthroscopy (NA) or conventional arthroscopy (CA) groups, including distracted (-D) and non-distracted (-nD) subgroups. The table summarises visualisation outcomes, surgical difficulty scores and procedure duration

Group	Visualisation of all predefined structures and palpation of both menisci	Medial meniscus visualisation			Lateral meniscus visualisation			Procedure time (mins)	Surgical difficulty score		
		100% visualised	50–99% visualised	<50% visualised	100% visualised	50–99% visualised	<50% visualised		1	2	3
NA (n = 20)	8 (40)	17 (85)	3 (15)	0 (0)	11 (55)	9 (45)	0 (0)	9.9 ± 1.9 (6.5–13.7)	16 (80)	4 (20)	0 (0)
NA-D (n = 10)	4 (40)	9 (90)	1 (10)	0 (0)	6 (60)	4 (40)	0 (0)	10.1 ± 1.8 (6.5–13.7)	7 (70)	3 (30)	0 (0)
NA-nD (n = 10)	4 (40)	8 (80)	2 (20)	0 (0)	5 (50)	5 (50)	0 (0)	9.8 ± 1.9 (6.9–13.7)	9 (90)	1 (10)	0 (0)
CA (n = 20)	6 (30)	12 (60)	8 (40)	0 (0)	12 (60)	8 (40)	0 (0)	11.5 ± 2.6 (6.3–15.3)	8 (40)	10 (50)	2 (10)
CA-D (n = 10)	3 (30)	6 (60)	4 (40)	0 (0)	6 (60)	4 (40)	0 (0)	12.0 ± 1.8 (9.6–15.3)	2 (20)	7 (70)	1 (10)
CA-nD (n = 10)	3 (30)	6 (60)	4 (40)	0 (0)	6 (60)	4 (40)	0 (0)	11.0 ± 3.0 (6.3–15.0)	6 (60)	3 (30)	1 (10)

Values are n (%) or mean ± SD (range). Results for NA are presented in shades of blue and results for CA in shades of green

### Dissection outcome measures

Craniolateral and craniomedial portals were positioned as planned in 35% and 88% of cases, respectively. Otherwise, portal sites were located peripherally within the patellar ligament (at a mean distance of 1.5 ± 0.5 mm [range 0.7–2.5] from the lateral or medial ligament edge). The long digital extensor tendon remained intact in all stifles, with a mean distance of 6.3 ± 1.2 mm (range 4.0–9.0) to the lateral portal site. The cruciate ligaments were intact, and femoral and tibial pins were consistently placed in the anticipated regions. In four (20%) specimens, tibial pins penetrated the medial collateral ligament.

**Figure 6:**
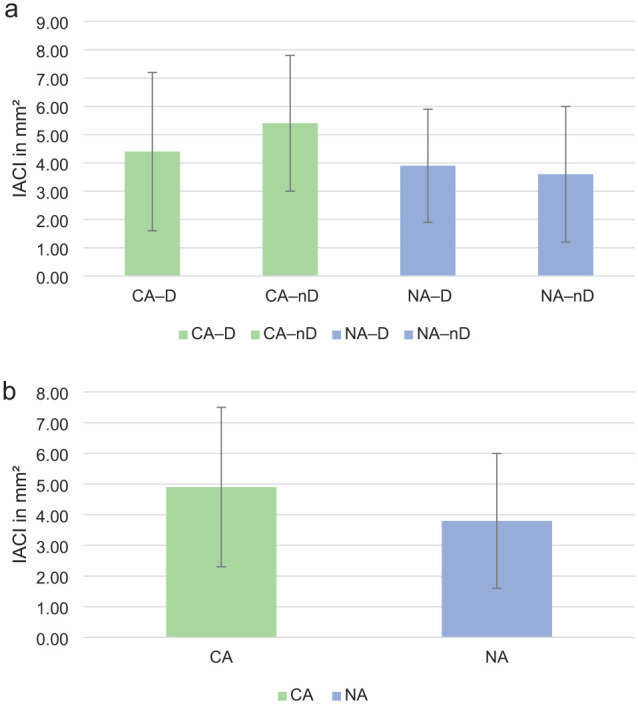
Comparison of absolute iatrogenic articular cartilage injury (IACI) (in mm^2^) resulting from conventional arthroscopy (CA) and needle arthroscopy (NA), performed with distracted (-D) and without joint distraction (-nD) in cadaveric feline stifle joints. (a) No significant differences were identified in absolute IACI across groups (CA-D: 4.4 ± 2.8 mm^2^, CA-nD: 5.4 ± 2.4 mm^2^, NA-D: 3.9 ± 2.0 mm^2^; NA-nD: 3.6 ± 2.4 mm^2^; *P* = 0.12–0.91). (b) Similarly, when subgroups were pooled, no significant differences were observed between CA and NA groups in absolute IACI (CA: 4.9 ± 2.6 mm^2^, NA: 3.8 ± 2.2 mm^2^; *P* = 0.11

IACI was detected in all specimens, with femoral cartilage affected in 100% of stifles, tibial cartilage in 45% and patellar cartilage in 50%. Most lesions were on the femoral condyles and trochlea (82.4% of the total IACI area), with a medial:lateral distribution ratio of 30:70. No differences were identified between subgroups regarding absolute IACI (CA-D: 4.4 ± 2.8 mm^2^, CA-nD: 5.4 ± 2.4 mm^2^, NA-D: 3.9 ± 2.0 mm^2^, NA-nD: 3.6 ± 2.4 mm^2^; *P* = 0.12–0.91) or percentage articular cartilage surface area IACI (CA-D: 1.0 ± 0.6%, CA-nD: 1.3 ± 0.6%, NA-D: 0.9 ± 0.4%, NA-nD: 0.9 ± 0.6%; *P* = 0.09–0.97). Similarly, absolute IACI (CA: 4.9 ± 2.6 mm^2^; NA: 3.8 ± 2.2 mm^2^) and percentage articular cartilage surface area IACI (CA: 1.1 ± 0.6%; NA: 0.9 ± 0.5%) did not differ significantly between the CA and NA groups (*P* = 0.11) (Figure 6). Cartilage injuries grossly appeared as partial-thickness lesions. Surgical difficulty was not associated with IACI.

## Discussion

Stifle arthroscopy using both CA and NA was successfully performed in feline cadavers without pre-existing pathology. NA improved surgical ease, shortened procedure duration and enhanced medial meniscus visualisation, without significantly reducing IACI. CA offered adjustable focus, subjectively better image quality and less arm movement, but was limited by cable bulkiness. NA allowed greater manoeuvrability and easier stifle manipulation, although with subjectively lower image quality.

Based on clinical experience and the study findings, the feline infrapatellar fat pad appears denser than in dogs, potentially obstructing arthroscopic access. To avoid fat pad obstruction and the need for debridement, portals were placed more proximally than is typically recommended in dogs.^26,27^ Nevertheless, fat pad obstruction remained common but was mostly manageable via distal retraction. The proximity of the arthroscope to femoral cartilage, along with the necessary fat pad manipulation, may increase the risk of IACI. However, no increase in periarticular or cartilage injuries was noted in the four stifles that required repeated portal placement. A more distal portal location with fat pad debridement should be evaluated, alongside assessing the safety of motorised shaver use in small joints.

Medial and lateral portals were placed peripherally within the patellar ligament in 12% and 65% of stifles, respectively. This distribution may reflect anatomical differences between cats and dogs, such as a wider patella and greater laxity of the feline patellofemoral joint.^28^ The five-fold higher incidence laterally likely resulted from efforts to avoid injury to the long digital extensor tendon. The tendon remained unharmed in all cases, suggesting that positioning lateral portals close to the patellar ligament should be avoided to prevent iatrogenic injury. Penetration of the medial collateral ligament by one-fifth of the tibial distraction pins was anticipated because of the chosen insertion site and is considered clinically insignificant.

Optimal irrigation pressures were higher for NA, which may be attributable to its subjectively lower image quality. Portal widening improved visualisation and periarticular fluid accumulation by creating additional egress sites. Previous feline studies provided no data on fluid ingress pressures,^15–17,19^ with the exception of one shoulder arthroscopy study that used a 50 mmHg pressurised cuff.^18^ In dogs, fluid ingress is typically regulated using pressure bags set between 150 and 300 mmHg or arthroscopic pumps calibrated to 30–80 mmHg,^10,29,30^ with one cadaveric study reporting 30 mmHg to be safe.^29^ Further studies are warranted to determine optimal and safe irrigation pressures for both canine and feline stifle arthroscopy.

Unrestricted manual limb manipulation clearly facilitated the procedure. Complete medial meniscus visualisation was achieved in 60% of CA stifles and in 90% and 80% of NA-D and NA-nD stifles, respectively. This contrasts with the lower medial meniscus visibility rates reported in a canine study using NA.^20^ The rates of complete lateral and medial meniscus visualisation were similar in the CA group, whereas the complete lateral meniscus visualisation rate in NA stifles (NA-D: 60%, NA-nD: 50%) was lower than for medial visualisation. This observation parallels the greater difficulty in evaluating the lateral meniscus with NA compared with CA in canine stifles,^20^ and may be related to differences in flexibility (0° vs 30°) and field of view (120° vs 80°) between needle and conventional arthroscopes, respectively. Improved lateral visualisation may be achieved by inserting the arthroscope through the medial portal to create a mirrored view, laterally distracting the joint and inducing cranial tibial thrust in unstable joints. Both previous feline stifle arthroscopy reports^15,16^ used 1.9 mm 30° arthroscopes. In one study, the menisci were evaluated as much as possible without iatrogenic injury,^16^ while in the other, the procedure was converted to an arthrotomy.^15^

Lower surgical difficulty scores in our study were, unsurprisingly, associated with improved overall and meniscal visibility as well as shorter procedure times across all groups. Mean arthroscopy times (NA: 9.9 mins, CA: 11.5 mins) were comparable to those reported in dogs (NA: 8 mins, CA: 12.5–15 mins).^20,31^ Distractor placement was quick and straightforward, requiring less than 1 min in all cases, and did not significantly affect overall procedure times. The shorter procedure duration associated with NA in the current and a previous canine study^20^ may be attributed to the improved manoeuvrability.

Extra-articular stifle distractors have been reported to improve visualisation^11–13^ and reduce IACI in dogs.^11^ However, in this study, no significant differences were observed between distracted and non-distracted groups. The distractor was subjectively considered helpful in approximately half of the cases across all groups, with no significant morbidity noted. The 1.6 mm pins, selected based on the distractor’s pinhole size, were consistently bent but did not fracture during distraction. Unlike in dogs,^12^ morbidities such as intra-articular pin placements were not observed, likely due to the use of specific anatomic landmarks.

IACI was observed in all specimens, exceeding the 93% incidence reported in dogs^10,31^ and matching the frequency observed in feline coxofemoral joints after NA.^19^ Although no statistical difference was observed, NA tended to cause less IACI than CA. This lack of significance may reflect a type II error due to insufficient power to detect small differences between groups. The total mean IACI area per joint (NA: 3.8 mm^2^, CA: 4.9 mm^2^), relative to joint size, was comparable to values reported in canine stifles after conventional 2.7 mm arthroscopy of CCL-intact stifles (range 5.2–5.9 mm^2^),^10,31^ and exceeded that in feline coxofemoral joints after NA (one to three lesions of 1–3 mm^2^ per joint).^19^ Cartilage injuries grossly appeared as partial-thickness lesions. However, this was not confirmed histopathologically, as fresh, unfrozen tissue would be required to ensure diagnostic accuracy.^32^ The long-term effects of partial-thickness cartilage lesions remain unclear, whereas full-thickness injuries are known to induce progressive osteoarthritis, and articular cartilage generally demonstrates limited regenerative capacity.^10,19^ Therefore, minimising IACI is essential and may be achieved through optimal limb positioning, appropriate equipment selection and refined technique. In dogs, IACI from CA was confined to the femoral articular surface,^10^ whereas in our study, all articular surfaces were affected. IACI was most prevalent on femoral condylar and trochlear cartilages, with over two-thirds located laterally. This distribution likely reflects the arthroscope insertion site. With femoral cartilage predominantly affected, proximal assessment should be performed cautiously, and alternative portal sites and techniques considered.

The relatively large size of current arthroscopic equipment raises questions about its benefit in small joints. Future studies should assess the clinical outcomes of arthroscopy vs arthrotomy to determine the safest and most effective approach for evaluating the feline stifle joint.

Limitations of this study include the use of cadaveric stifles, which may not fully reflect in vivo conditions – particularly regarding musculotendinous and cartilaginous structures, joint space, range of motion and distraction effectiveness.^19,32^ Stable joints may have reduced visualisation, as CCL-deficient stifles reportedly provide improved views of the caudal joint space with cranial tibial thrust.^12^ Clinically, however, CCL deficiency may impair visibility due to joint capsule and synovial thickening.^19^ Intact CCLs may have limited distraction effectiveness, although a separate feline cadaver showed no difference in arthroscopic feasibility or distraction performance with severed CCLs. Limited distraction and the increased susceptibility of cadaveric cartilage to injury may have contributed to the higher IACI scores, although softened cartilage in osteoarthritic joints is similarly predisposed to injury.^32^ Although cartilage lesions are generally distinguishable visually,^31^ a further limitation remains the potential for inaccuracies in assessing lesion origin (native vs iatrogenic) and depth. Finally, apart from dissection outcome measures, blinding due to the nature of the procedures was not feasible, which may have introduced observer or performance bias.

Further studies are warranted to evaluate the impact of medial and lateral joint distraction, arthroscope insertion site, CCL deficiency and other factors on intra-articular visibility, surgical difficulty, IACI and the clinical application of feline stifle arthroscopy.

## Conclusions

Both NA and CA were feasible for use in feline cadaveric stifle joints, with NA offering easier handling, shorter procedure times, improved medial meniscus visualisation and a tendency towards less IACI.

Portals were placed laterally and medially to the patellar ligament with minimal morbidity. The lateral portal commonly penetrated the patellar ligament, likely due to attempts to avoid the lateral extensor tendon, which consistently remained out of range of the portal. A balanced portal trajectory is therefore needed to prevent injury to either structure.

Proximal portal placement generally allowed adequate visualisation without fat pad debridement; however, the accumulation of IACI near the arthroscope insertion site suggests the need to explore alternative portal sites and fat pad debridement.

The use of an extra-articular stifle distractor did not significantly improve procedural outcomes. IACI was comparable to that observed in dogs,^10,31^ and may be reduced by refining portal positions, fat pad debridement and surgical technique.

Our findings suggest that arthroscopy is a viable tool for feline stifle joint procedures, with NA emerging as a promising alternative to CA in terms of feasibility and efficiency. Further studies are warranted to assess its clinical application and long-term outcomes.
